# Echoes of Autism? Inhaled Ultrafine Particles and Brain Changes in Mice

**DOI:** 10.1289/ehp.122-A250

**Published:** 2014-09-01

**Authors:** Carol Potera

**Affiliations:** Carol Potera, based in Montana, has written for *EHP* since 1996. She also writes for *Microbe*, *Genetic Engineering News*, and the *American Journal of Nursing*.

Epidemiological evidence has raised the possibility that exposure to air pollution could be a risk factor for autism spectrum disorders (ASDs)^1^^,^^2^^,^^3^ and schizophrenia.^4^ For example, one recent study of children living in California reported that those exposed to the highest levels of traffic-related air pollution during pregnancy and in the first year of life were twice as likely to develop ASDs as those exposed to the lowest pollutant levels.^2^ In this issue of *EHP*, researchers present the results of laboratory studies investigating a potential explanation for findings such as these.^5^

The team, led by Deborah Cory-Slechta of the University of Rochester Medical Center (URMC), found that exposure to ultrafine particles early in life produced brain changes in mice suggestive of those in humans with ASDs and schizophrenia.^5^ Notably, the brains of exposed male mice showed enlarged lateral ventricles, a condition called ventriculomegaly. “That’s alarming, because ventriculomegaly in humans has behavioral consequences” including ASDs^6^ and schizophrenia,^7^ says Cory-Slechta.

**Figure:**
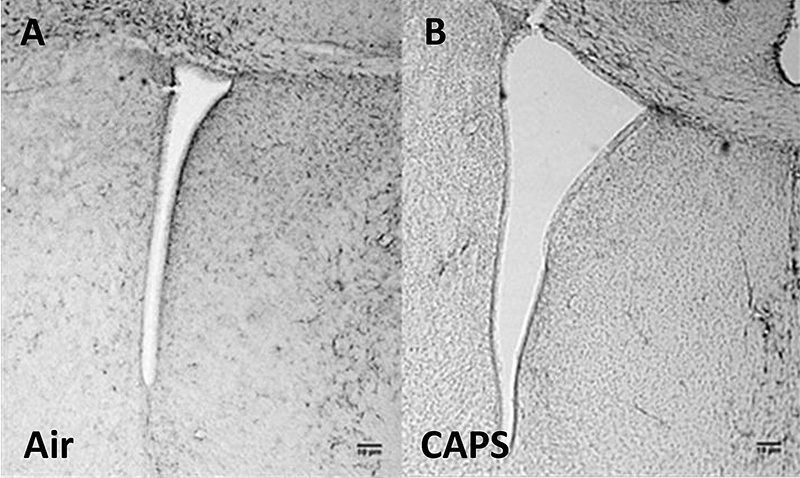
Images of lateral ventricles from control (A) and exposed (B) mice at postnatal day 14 show enlargement resulting from exposure to particulate matter (identified here as “concentrated ambient particles,” or CAPs). Image: Allen et al. (2014)^5^

The researchers exposed mice to ultrafine particles (less than 100 nm in diameter) at concentrations comparable to those in large cities during rush hour traffic. Exposures occurred for 4 hours per day on postnatal days 4–7 and 11–13. The first two weeks of life for mice are equivalent to the third trimester in human pregnancy and thus are a critical time for brain development, Cory-Slechta says.

One mouse group was examined 24 hours after stopping exposure. Females’ brains showed evidence of inflammation, and males’ lateral ventricles were 2–3 times larger than normal. These changes persisted in females examined at postnatal day 55 (adolescence for mice) and males examined at postnatal day 270 (later adulthood), suggesting permanent brain damage.^5^

“We never considered ultrafine particles to be overtly toxic to the brain. It’s time to consider that possibility,” says first author Joshua Allen, an assistant research professor at URMC. In other mouse studies, the URMC team has reported that inhaling ultrafine particles was also associated with changes in behavior^8^ and memory^9^ indicating impaired neurodevelopment.

In the United States, the estimated incidence of ASDs in 8-year-olds is 1 in 42 for boys, and 1 in 189 for girls.^10^ According to the authors, early exposure to ultrafine particles in early childhood may turn out to be a risk factor for ASDs. Importantly, however, although the findings are suggestive, it is premature to conclude from these results that air pollution causes ASDs in people.

What the new results do provide is novel information on the potential effects of ultrafine particles on the developing nervous system, says Lucio Costa, a professor of toxicology at the University of Washington in Seattle, who was not involved in the study. They also add to the evidence suggesting a causal association between exposure to air pollution and neurodevelopmental abnormalities. “This area of research warrants further studies to probe such associations and define their underlying mechanisms,” Costa says.
